# Report of Three Cases Illustrating the Correlation Existing between Erysipelas, Diphtheria (and Hospital Gangrene?)

**Published:** 1864-06

**Authors:** H. S. Chalmers


ART IV.—*Report of three cases illustrating the Correlation existing between Erysipelas, Diphtheria (and Hospital Gangrene?)* By Assistant Surgeon H. S. CHALMERS.

Case 1.—Private George T. Weightman, Co. "A," 11th Va. Vols., admitted in General Hospital, Lynchburg, May 10th, 1862, with gun-shot wound of hand and fore-arm, received May 5th. He is a robust, healthy man, age 23, by occupation before enlistment a merchant. The ball entered on the dorsal aspect of the hand, between the metacarpal bone of the middle and third fingers, passed longitudinally through the carpus, and made its exit on the inner aspect of the fore-arm, two and a half inches from the wrist joint. The lower extremity of the radius is fractured, the ulna entire. The patient was treated in private quarters and the symptoms from the date of admission, as follows:

May 10th.—There is active inflammation in the limb, with severe pain, heat and swelling; sthenic fever, with pulse about 100. Ordered rest, light diet, and cold-water dressing, the arm supported on a pillow.

From the 11th to 15th, the inflamation has progressed to imperfect establishment of suppuration. The swelling has extended to the shoulders, and the pain is very severe. The pus is not healthy, is very abundant but mixed with sanious fluid, patient's strength and appetite are much improved, his fever is asthenic or hectic, pulse 120. Treatment ordered is wine, generous diet, warm water and oil silk dressing.

During the 16th and 17th the inflammation rapidly became erysipelatous; patient suffered with rigors and great debility and nervous disturbance. Treatment consisted of tincture ferri chloridi with quinine, freely administered; wine and generous diet continued; and, locally, solution of nitrate of silver, from twenty grains to one ounce.

On 18th, false membrane formed on both wounds so dense as to arrest the discharge entirely. Free incisions were made to relieve tention, and on the edges of these the membrane rapidly formed, also on the abraded surfaces left by broken blebs.

On the 25th the erysipelas began to subside and healthy suppuration to be established; the patient convalesced slowly and was discharged from treatment July 11th.

The limb preserved the motions of pronation and supination; the wrist joint was completely anchylosed; enough control over the thumb and fore-finger to grasp a pen lightly.

On the 15th May, the day on which the erysipelas appeared in the injured limb, a child one a half years old, who was in the same room was taken sick and came under care of a private physician. 18th—I was invited to examine the child, and found the case to be one of diphtheria, the membrane lining the faucis upper part of pharynx, etc. The child died on the 19th.

On the 20th, a married woman, the sister of my patient, and who spent most of her time in the same room, complained of sore throat, and on examination I found several small patches of false membrane on the back of the pharynx with the glairy mucus discharge from the adjacent mucous membrane and from the nares, which is characteristic of the disease. She was relieved in two or three days.

Case 2.—Private Wm. G. Newman, Co. "H," 58th Va. regiment, was admitted in the General Hospital, Lynchburg, August 11th, 1862, with amputation of thigh at lower third. Was a healthy man, age 34, occupation before enlistment a farmer. He had been wounded August 9th, a fragment of shell striking him about the knee. Amputation was performed on the 10th, and the patient sent on to Lynchburg, a distance of seventy miles. Up to August 18th the patient did well. The stump remaining in a healthy condition, and the angles of the flaps (the operation by the flap method) uniting by the first intention. There were several cases in the same ward of wounds effected with ulcerative phagedæna, but no cases of gangrene in mass. On the 19th August the flaps began to slough, the patient presenting at the same time the usual symptoms of hot, dry skin; dry, red tongue; frequent pulse, thirst, loss of appetite, etc. Active supporting treatment was used, with, locally, creasote lotion, and the yeast and charcoal poultice. The sloughing continued to advance till the 30th, when the sloughing was arrested. There was no further sloughing for ten days, but the stump remained in an indolent condition; the granulations were large, flaccid and of a purplish hue; there was no laudable pus, but an abundant exudation of sanious fluid mixed with pus. The general condition of the patient improved somewhat.

On the 9th September the gangrene was set up afresh, and when giving the patient fluids they were observed to return by the nares; he had not complained of sore throat. An examination revealed the fact that the fauces and pharynx, as far back as could be seen, were covered with false membrane. The membrane was thin and easily removed from the surface.

On the 10th and 11th, it had advanced till it could be seen at the anterior nares. There was also an imperfect membrane formed on the track of the cauteries which were applied to the stump. Patient died on the night of September 27th.

Case 3.—Lieut. J. P. Canterbury, of Alabama volunteers, admitted in General Hospital, Lynchburg, November 20th, 1862, with gun-shot wound of shoulder, received September 17th. The ball entered on the front aspect of the arm, just below the joint, and passed downwards and backwards on the outer side of the bone. The shaft of the humerus was splintered, but its continuity not destroyed. At the time of admission all inflammation had subsided. The cicatrizing wound was attempting to close around a small mass of protruding granulations, indicating the presenee of necrosed bone which could be readily felt with a probe, but was not detached.—There was abundant formation of callus around the injured portion of bone, and a free discharge of healthy pus from the wounds, both of entrance and exit.

Dec. 20th.—The patient was seized with rigors, pain in epigastric region, nausea and high fever.

On 22d, became delirious, and facial erysipelas is well developed.

On the 24th, the erysipelas has subsided down on the neck and chest, and attacked the wounded limb. In the wounded arm the form of the disease is phlegmonous. He has had several slight convulsions which come on whenever it is attempted to move him in bed.

On the 25th, there was formation of diphtheritic membrane on roof of mouth, fauces and pharynx, with difficulty of swallowing, and the return of fluids by the nose. The patient became comatose on the 27th, and died during the night following. At the time this case occurred two-thirds of the wounds in the wards were in a more or less gangrenous condition.

